# Predictors of consistent condom use based on the Information-Motivation-Behavioral Skills (IMB) model among female sex workers in Jinan, China

**DOI:** 10.1186/1471-2458-11-113

**Published:** 2011-02-17

**Authors:** Hua Zhang, Meizhen Liao, Xijuan Nie, Rongjian Pan, Chuangxin Wang, Shiman Ruan, Changqing Zhang, Xiaorun Tao, Dianmin Kang, Baofa Jiang

**Affiliations:** 1 Department of Epidemiology and Biostatistics, School of Public Health, Shandong University, Jinan, Shandong Province 250012, China; 2 Institute for AIDS/STD Control and Prevention, Shandong Province CDC, Jinan, Shandong Province 250014, China; 3 Institute for AIDS/STD Control and Prevention, Licheng District CDC, Jinan, Shandong Province 250100, China; 4 Institute for AIDS/STD Control and Prevention, Jinan City CDC, Jinan, Shandong Province 250000, China

## Abstract

**Background:**

Female commercial sex workers (FSWs) are at high risk of human immunodeficiency virus (HIV) transmission in China. This study was designed to examine the predictors of condom use with clients during vaginal intercourse among FSWs based on the Information-Motivation-Behavioral Skills (IMB) model and to describe the relationships between IMB model constructs.

**Methods:**

A cross-sectional study was conducted in Jinan of Shandong Province, from May to October, 2009. Participants (N = 432) were recruited using Respondent-Driven Sampling (RDS). A self-administered questionnaire was used to collect data. Structural equation modeling was used to assess the IMB model.

**Results:**

A total of 427 (98.8%) participants completed their questionnaires. Condom use was significantly predicted by social referents support, experiences with and attitudes toward condoms, self-efficacy, and health behaviors and condom use skills. Significant indirect predictors of condom use mediated through behavioral skills included HIV knowledge, social referents support, and substance use.

**Conclusions:**

These results suggest that the IMB model could be used to predict condom use among Chinese FSWs. Further research is warranted to develop preventive interventions on the basis of the IMB model to promote condom use among FSWs in China.

## Background

Since the 1980 s the number of female commercial sex workers (FSWs) has markedly increased in China [1]. The number of women engaging in commercial sex is estimated to be 4 to 10 million [1,2]. Commercial sex plays a critical role in heterosexual transmission of human immunodeficiency virus (HIV) in China [1]. Approximately 127,000 female sex workers and their clients were living with HIV/AIDS, accounting for 19.6% of the total number of estimated HIV cases at the end of 2005 [3]. Heterosexual sexual transmission among cumulative HIV infection increased from 10.7% in 2005 to 37.9% in 2007, accounting for 44.7% of the 50,000 estimated new infections in 2007 [4]. Heterosexual transmission of HIV through contact with FSWs is of particular concern [2,4,5]. Data from national sentinel surveillance indicated that the prevalence of HIV among FSWs averaged 1%, with the highest rates at 10% in several sites [6].

Sex trade is not accepted by Chinese ethical standards and there is a substantial stigma to FSWs. They are discriminated and marginalized in China [7]. In addition, the sex trade in China is clandestine due to unfavorable laws that impose fines and incarceration on FSWs and their clients if arrested. However, some women become FSWs possibly due to being attracted by the relatively high income. According to previous reviews, most FSWs in China are young, mobile, less educated, and some FSWs have also reported substance abuse [1,5]. They often trade sex in entertainment establishments/service sectors (*e.g*. dance hall, night clubs, restaurants, barber shop, and sauna parlors), are less likely to use condoms, and have high rates of sexually transmitted infections (STIs) [1,5].

Given lack of effective vaccine or treatment of HIV, changing an individual's risk behaviors is crucial to prevent the spread of HIV. Consistent and correct condom use seems to be the most effective form of HIV prevention among FSWs. However, a recent review reported rates of consistent condom use with clients among FSWs in China of between 13% and 54% [1]. Recent national comprehensive surveillance data showed that 60% of FSWs in China do not use condoms regularly [4]. It would be imperative to determine predictors of consistent condom use among FSWs to develop effective interventions for the prevention of HIV spread in China.

The Information-Motivation-Behavioral Skills (IMB) model developed by Fisher and his colleagues was designed to predict HIV preventive behavior and necessary elements HIV prevention intervention [8,9]. The conceptualization of the IMB model holds that HIV prevention information, motivation, and behavioral skills are the fundamental determinants of HIV preventive behavior. The model assumes that HIV prevention information and motivation affect risk-reduction behavioral change largely through behavioral skills. Information and motivation may also have direct effects on HIV preventive behavior, particularly when the behavior requires relatively uncomplicated behavioral performances. Furthermore, HIV prevention information and motivation are regarded as independent constructs, demonstrated by well-informed people who are not motivated to practice preventive behaviors, and by individuals who are motivated to practice preventive behaviors but are not well informed [9,10].

The IMB model has been successfully tested in a variety of risk groups [11-20]. The constructs of the IMB model are regarded as highly generalizable determinants of HIV preventive behaviors in any population [9]. The current study used the IMB model as the theoretical framework to determine predictors of condom use among FSWs. According to the IMB model, motivation to engage in HIV preventive behaviors includes three factors that affect motivation to act: attitudes, social norms, and perceptions of personal vulnerability. Social norms are hypothesized to be a function of a person's perceptions of what social referents think should be done regarding the behavior *multiplied by *the person's motivation to comply with social referents [9,21]. The motivation to comply represents the extent to which a person wants to act as a reference group member and is assessed by the importance of social referents' belief [21]. Social norms, the perceptions of social referents support, and motivation to comply can affect HIV prevention intentions and behaviors [9]. In the literature, social referents support for condom use was used as one of indicators of motivation [10,15-17,20]. Pro-condom norms, which were assessed by the importance of the approval of referents in using condoms, significantly predicted condom use and self-efficacy in adolescent substance use study [18]. To examine the role of the motivation to comply within the context of the IMB model among FSWs, it has been hypothesized that the motivation to comply and the perceptions of social referents support were two indicators of social norms. Fisher holds that specific social referents support is salient for HIV preventive behaviors within a given population, and that perceptions of social norms for HIV prevention behaviors should be adjusted to increase motivation to perform such behaviors [9]. In the current study, peers, employers, and clients were regarded as social referents because their support has been demonstrated to be significantly associated with consistent condom use among FSWs [22-28].

Fisher has identified a broad range of behavioral skills that are assumed to be necessary for HIV preventive behaviors [29]. "Universal" HIV prevention behavioral skills include the objective ability to perform such acts and a sense of self-efficacy for doing them, such as skills to negotiate HIV preventive behavior, to engage in public behaviors such as HIV testing and condom purchasing, and to avoid drinking or drug use before sex [9,16,30]. Bandura holds that one must possess these behaviors and self-efficacy to engage in HIV prevention [31]. Measures of self-efficacy for performing a behavior have been commonly used in previous studies [10,16,17,20]. Self-efficacy may not completely represent skill performance as much as personal acceptance for practicing a skill and engaging in the public behaviors. Two indicators of behavioral skills have been assessed: condom self-efficacy and condom use skills among adolescent population and mentally ill adults [18,19]. The two studies demonstrated that condom application skills were significantly associated with certain factors (*e.g*., condom attitudes), but were not related to condom use. In the current study, measures of both self-efficacy and health behaviors and condom use skills were used as indicators of behavioral skills.

In addition, many studies have reported that substance use is the greatest risk factor for HIV/STI infection and for inconsistent condom use among FSWs [6,32-35]. In China, sentinel sites with FSWs who use drugs had higher HIV rates than those sites with only non-drug-using FSWs [36]. Fisher also holds that "universal" HIV prevention behavioral skills may be relevant to substance use [9]. Therefore, substance use status was added to this model.

The current study aimed to explore predictors of condom use during vaginal intercourse between FSWs and clients within the context of the IMB model and to examine the relationships between these constructs of the model. It was hypothesized that information, motivation, and substance use would predict both health behaviors and condom use skills and self-efficacy, and these indices of behavioral skills would, in turn, predict condom use.

## Methods

### Study site

The study was conducted from May to October 2009 in Jinan, the capital city of Shandong Province, with about 3 million of permanent residents and 1.5 million of migrants. The majority (98.2%) of the population in Jinan are Han Chinese.

A total of 2,167 HIV/acquired immunodeficiency syndrome (AIDS) cases have been reported in Shandong by the end of 2008; heterosexual transmission was the major transmission route [37]. In Jinan, it is estimated that 50,000-100,000 employees work in entertainment establishments and about one-third of them are sex workers [38]. Recent surveillance results showed that 30.2% of HIV-positive cases in Jinan were infected through heterosexual transmission [39].

### Recruitment of seeds and study participants

Respondent-Driven Sampling (RDS) was used to recruit participants. RDS is an adaptation of chain-referral sampling and a suitable sampling method for hidden populations, which can provide relatively unbiased and representative population-based estimates [40,41].

The selection criteria for eligible FSW seeds and participants were defined as "a female over the age of 18 who has exchanged sex for money and has lived in Jinan in the past month and is not inebriated at the interview." Each seed or participant was asked to recruit no more than three peers.

Six seeds were initially selected and told how to refer other eligible FSWs. Each seed was given three uniquely coded coupons to refer their peers. Coupons were given to participants until approximately 350 participants were recruited, to obtain a target sample size of approximately 400 FSWs. Seeds and their recruits were given an incentive package including 50 Yuan (Ұ 50, equal to U.S. $ 7.32), HIV prevention pamphlets and four boxes of condoms for successfully participating, plus an additional 20 (U.S. $ 2.93) for recruiting a FSW. A total of 957 coupons were given out to 319 FSWs, and 9 females refused to participate in the study.

A structured questionnaire was used to collect data administered by trained female interviewers. An informed consent form was read to each candidate, describing the potential risks and benefits of the research. Interviewers explained to every candidate that voluntary participation, anonymity, and confidentiality were ensured. The participants privately completed a self-administered questionnaire. For participants with limited literacy (about 2.1%), only one interviewer who provided assistance as needed was allowed to stay with the participant, and the interviewer read each question and response options from the questionnaire, while the participant marked the response on her own questionnaire. A total of 432 FSWs were recruited. The numbers of missing data for all variables ranged from 3 to 1. Cases with any missing data were excluded, resulting in a sample size of 427. The study protocol was approved by the Ethics Review Committee of Shandong University.

### Measures

A structured questionnaire was developed to collect data on demographics, such as age, ethnicity, education, permanent residence, marital status, and the constructs of the IMB model. The IMB model constructs included HIV/AIDS prevention information, motivation, behavioral skills, and prevention behaviors. Each measure of the IMB model constructs is described below.

#### Information

*Information* was measured with 18 items scaled 0-1 that assessed FSW knowledge about HIV/AIDS [42-44]. One indicator containing 11 items was related to HIV transmission (*e.g*., Do you think people can catch AIDS by blood transfusion?); another one containing 7 items was related to HIV prevention (*e.g*., Do you think correctly using a condom during every sexual intercourse can prevent HIV infection?). Participants were asked whether the statements were true or false, or unknown. "Don't know" responses were coded as incorrect answers. Cronbach's alpha coefficient for the 11 HIV transmission related items and 7 HIV prevention related items was 0.78, and 0.81, respectively.

#### Motivation

*Experiences with and attitudes toward condoms* were assessed with 7 true or false questions. Typical items included: "Condom use causes too much trouble" and "Condom use is safer than other methods." The number of answers indicating a positive attitude served as an indicator of experiences with and attitudes toward condoms. The composite score ranged form 0 to 7. Cronbach's alpha coefficient for the 7 items was 0.80.

*Social referents support* contained three indicators, assessed with nine items from Bazargan [15], Yang [22], Kerrigan [23], and Wang [26]. In the current study, peers, employers, and clients were regarded as social referents. Typical items included "how much would your employer approve or disapprove of your use of condoms with clients?" and "how much would your employer approve or disapprove of you talking about safe sex with clients?" Each item was scored 1 (disapprove strongly) to 5 (approve strongly). The means of the responses associated with the three items for each social referent were calculated yielding three items as indicators of social referents support. Cronbach's alpha coefficient ranged from 0.75 to 0.83 for three subscales of social referents support.

*Motivation to comply* was measured by three items indicating the importance of the approval of peers/employers/clients in using condoms [21]. Responses ranged from 1 (extremely unimportant) to 5 (extremely important). Cronbach's alpha coefficient was 0.68.

*Perceived risk* was indicated by three items that asked (a) the extent to which the FSW was about getting AIDS on a scale ranging from 1 (not at all worried) to 5 (extremely worried); (b) her risk of getting AIDS on a scale ranging from 1 (very low) to 5 (very high); and (c) her risk of getting a sexually transmitted infection on a scale from 1 (very low) to 5 (very high) [45]. Cronbach's alpha coefficient was 0.56.

#### Substance use

*Substance use* was measured by two items. Item 1 asked if the FSW has used non-injection drugs. Item 2 asked how many times the FSW drunk alcohol in the last week. Non-injection drugs use was scored 0 and no drugs use was scored 1. No alcohol use was scored 5; the frequencies of alcohol use that ranged from 1 to 2, 3 to 4, 5 to 6, and more than 7 were scored 4, 3, 2, and 1, respectively. Higher scores indicated lower frequencies of alcohol use in the last week. Another item, indicating whether the FSW had ever used injection drugs, was eliminated because answers revealed that only 0.7% of FSWs reported that they had ever used injection drugs. Cronbach's alpha coefficient for the two items was 0.61.

#### Behavioral skills

*Health behaviors and condom use skills* were assessed by four indicators [9,11]. For instance, "Have you been HIV tested in the last year?" and "Was condom always available?" Condom use skills were measured as a composite score, which is the sum of the 5 item scores. The 5 items were measured as dichotomous variables (0 = no, 1 = yes), asking whether woman had performed correct condom use: (a) checking the expiration date and opening the condom package without tearing the condom; (b) pinching the tip of the condom to remove air; (c) putting the condom on the client before intercourse; (d) the condom being rolled to the base of the erect penis; (e) after ejaculation, holding the rim of the condom and pulling the penis out before it got soft [22]. Cronbach's alpha coefficient was 0.76 for the 5 items.

*Self-efficacy* in practicing HIV prevention was assessed by 8 items [11,22,46]. Three items were used to measure self-efficacy for condom negotiation skills. Typical items included "Can you discuss safe sex with your clients before sex?", and "Can you persuade your clients to use a condom if he is unwilling to use it?". Cronbach's alpha coefficient for the 3 items was 0.83. A composite score was created using the mean of three items that were scored 1 (very difficult) to 5 (very easy). Three items were used to measure self-efficacy about refusing unprotected sex (*e.g*., "Can you refuse to have sex with client when he does not use a condom by extra money?") Cronbach's alpha coefficient for the 3 items was 0.85. Similarly, a composite score was created using the mean of three items that were scored 1 (very difficult) to 5 (very easy). Self-efficacy for acquiring HIV prevention information and self-efficacy for avoiding drinking or drug use before sex was respectively measured by one item that was scored from 1 (very difficult) to 5 (very easy).

#### Percent condom use

The percentage of condom use was used as the dependent variable in the study. It was calculated by the times of condom use divided by the number of vaginal intercourse during vaginal intercourse with clients in the last month.

## Data analysis

Descriptive statistics, such as means, standard deviations, frequencies, and percentages were reported. The hypothetical IMB was examined by structural equation model (SEM) using the LISREL [47]. SEM compares a proposed hypothetical model elucidating relationship with a set of actual data. The closeness of the variance-covariance matrix implied by the hypothetical model to the empirical variance-covariance matrix is evaluated through goodness-of-fit indices, including maximum likelihood chi-square values/degrees of freedom ratio, the comparative fit index (CFI), the root mean square errors of approximation (RMSEA), and the non-normed fit index (NNFI). Good fit (a chi-square valueless than twice the degrees of freedom in the model; CFI≥0.95; NNFI≥0.95; RMSEA < 0.06) was used to justify interpretation of parameters [15,47,48].

Confirmatory factor analysis (CFA) was conducted to examine the factor structure (measurement model) and the relationships among all the latent variables and manifest variables. Once the factor structure was confirmed, a path model was performed to examine the predicting effects of all study variables on condom use and the effects of HIV preventative information and motivation on behavioral skills. In an effort to generate a parsimonious model, non-significant paths were gradually dropped until only significant paths remained. In the trimming of the model, we first reviewed the path coefficients. One non-significant path was eliminated, and the difference *x*^2 ^was evaluated at each step. Non-significant improvement in the *x*^2 ^value would indicate that it was acceptable to trim this path.

## Results

Table 1 presents sample demographics. Mean age of the sample was 29.6 years (SD = 7.4), 66.7% were older than 25 years, 27.4% had first lifetime sex at <18 years of age, 99.1% was Han in ethnicity, 66.0% received middle school education or less, and 32.6% were divorced or widowed. Most of the participants (81.5%) reported having noncommercial steady sexual partner(s) and 45.7% had no children. The mean number of clients was 4.5 (SD = 3.4) in the last week and the mean number of clients in the last month was 14.3 (SD = 8.2). The mean number of FSWs which the participants knew in Jinan was 12.5 (SD = 7.9). The majority (82.7%) earned more than 2,000 Yuan (or approximately U.S. $ 300) per month, 13.6% satisfied the client's desire for sex without using a condom if extra money was provided, and 63.9% reported ever had STD-related symptoms in the past year.

**Table 1:** Sociodemographic characteristics of female sex workers (N = 427)

Characteristics	Frequency	Percentage (%)
Age (years)		
18-20	40	9.4
21-25	102	23.9
26-53	285	66.7
Ethnicity		
Han	423	99.1
Other	4	0.9
Education level		
Primary school or lower	56	13.1
Middle school	226	52.9
High school or higher	145	34.0
Registered permanent residence		
Jinan city	56	13.1
Other city in Shandong	145	34.0
Outside Shandong	226	52.9
Martial status		
Single	103	24.1
Cohabitation	68	15.9
Married	117	27.4
Divorced or widowed	139	32.6
Number of children		
0	195	45.7
1	207	48.5
≥2	25	5.8
Monthly in come (Yuan Ұ)		
≤2000	74	17.3
> 2000	353	82.7
Age at sex debut (years)		
≥18	310	72.6
< 18	117	27.4

The mean of the percent of correct responses to HIV prevention knowledge was only 45.7% and the mean of the percent of correct responses to HIV transmission knowledge was 61.2%. 47.1% of the women reported using condoms with clients every time during vaginal intercourse in the last month. In addition, 27.0% of the sample's clients supported condom use with FSWs during sexual intercourse. Only 19.4% of the women were tested for HIV in the past year.

### Confirmatory factor analysis

**Figure 1:**
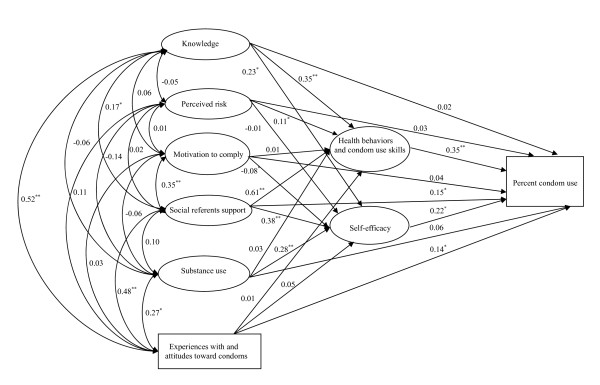
**The full predictive IMB model**. Structural equation model depicting regression paths in the IMB model (N = 427). Large circles represent latent variables; rectangles represent single-item indicators. Single-headed arrows represent regression coefficients; double-headed arrows represent correlations. Regression coefficients are standardized (*p≤0.05, **p≤0.001).

The means, standard deviations, ranges, and factor loadings for constructs of the model are shown in Table 2. As shown in table 2, all factor loadings were significant (*p*≤0.05). The fit statistics for the saturated model were acceptable: *x*^2 ^=386.61, 197 *df*, CFI = 0.95, RMSEA = 0.05, NNFI = 0.90. The full model included all paths appears in Figure 1. Measured variables representing latent constructs are in rectangles and the multiple indicator latent variables are in ovals. The full model predicted 42% of the variance for condom use, 62% of the variance for health behaviors and condom use skills, and 45% of the variance for self-efficacy.

**Table 2:** Summary statistics and factor loadings in confirmatory factor analyses

	M	SD	FL*
Knowledge			
Prevention knowledge (% correct)	45.7	25.7	0.69
Transmission knowledge (% correct)	61.2	21.4	0.66
Prevention Motivation			
Perceived risk (1-5)			
Fear about getting HIV	4.3	1.1	0.45
Chance of getting HIV	2.1	1.3	0.46
Chance of getting STDs	3.3	1.1	0.41
Motivation to comply (1-5)			
Motivation to comply with partner	3.9	1.4	0.62
Motivation to comply with employer	3.3	1.5	0.93
Motivation to comply with client	3.2	1.6	0.62
Social referents support (1-5)			
Partner support	4.3	0.7	0.67
Employer support	3.5	0.8	0.80
Client support	3.3	0.9	0.65
Experiences with and attitudes toward condoms (0-7)	4.3	2.0	-
Substance use			
Alcohol use (1-5)	2.7	1.5	0.52
Non-injection drugs use (0-1)	0.9	0.3	0.47
Health behaviors and condom use skills			
Condom accessibility (1-5)	4.6	0.7	0.54
Condom use skills (1-5)	3.5	1.5	0.60
HIV testing (0-1)	0.2	0.4	0.59
Gynecological examination (0-1)	0.6	0.5	0.52
Self-efficacy (1-5)			
Condom negotiation skills	3.1	0.6	0.63
Acquire HIV prevention information	3.4	1.1	0.48
Refusal of unprotected sex	3.1	0.9	0.85
Avoid drinking or drug use before sex	3.7	1.3	0.44
Percent condom use	0.9	0.2	-

M = mean; SD = standard deviation

*FL = factor loadings, all significant≤0.001

Table 3 reports the bivariate correlations among constructs in the model without any directionality of influence among them. The correlations show that condom use was associated with social referents support, experiences with and attitudes toward condoms, substance use, health behaviors and condom use skills, and self-efficacy.

**Table 3:** Correlation among model variables

		I	II	III	IV	V	VI	VII	VIII	IX
I	Knowledge	1.00								
II	Perceived risk	-0.05	1.00							
III	Motivation to comply	0.06	0.01	1.00						
IV	Social referents support	0.17*	0.02	0.35^++^	1.00					
V	Experiences with and attitudes toward condoms	0.52^++^	0.11	0.03	0.48^++^	1.00				
VI	Substance use	-0.06	-0.14	-0.06	0.10	0.27 ^+^	1.00			
VII	Health behaviors and condom use skills	0.43^++^	0.15*	0.09	0.49^++^	0.47^++^	0.41^++^	1.00		
VIII	Self-efficacy	0.27^+^	0.08	-0.20^+^	0.41^++^	0.41^++^	0.29^+^	0.57^++^	1.00	
IX	Percent condom use	0.03	0.02	0.09	0.37^++^	0.33^++^	0.24^+^	0.51^++^	0.55^++^	1.00

*p < 0.05; ^+ ^*p *< 0.01; ^++ ^*p *< 0.001

### Path models

**Figure 2:**
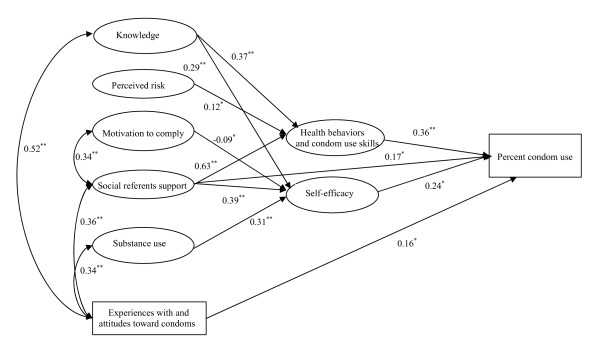
**The final predictive IMB model structural equation model**. Structural equation model depicting significant regression paths in the IMB model (N = 427). Large circles represent latent variables; rectangles represent single-item indicators. Single-headed arrows represent regression coefficients; double-headed arrows represent correlations. Regression coefficients are standardized (*p≤0.05, **p≤0.001).

The final IMB model is depicted in Figure 2. The fit indices for the trimmed model were acceptable: *x*^2 ^=405.50, 206 *df*, CFI = 0.97, RMSEA = 0.05, NNFI = 0.95. The explained variances for condom use, health behaviors and condom use skills, and self-efficacy were 38%, 57%, and 43%, respectively.

As shown in Figure 2, significant predictors of condom use were social referents support, experiences with and attitudes toward condoms, self-efficacy, and health behaviors and condom use skills. Health behaviors and condom use skills was significantly predicted by HIV knowledge, perceived risk, and social referents support. Significant predictors of self-efficacy included HIV knowledge, motivation to comply, social referents support, and substance use.

The indirect effects on condom use as reported in the trimmed model were also examined. Variables that indirectly affected condom use through health behaviors and condom use skills or self-efficacy variables included HIV knowledge (*p*≤0.001), social referents support (*p*≤0.001), and substance use (*p*≤0.05).

## Discussion

The results of this research demonstrate the applicability of the IMB model in predicting HIV/STI risk behaviors among Chinese FSWs. The results of this study show that FSWs who possessed higher levels of social referents support, health behaviors and condom use skills, self-efficacy, and positive experiences with and attitudes toward condoms were more likely to use condoms with clients during vaginal intercourse.

In the current study, the direct effect of HIV knowledge on condom use was not significant. These results are consistent with prior research that used the IMB model with gay men and heterosexual college students [11], women of low-income housing [14], juvenile offenders [20], and substance use adolescents [18]. Bandura and Fisher hold that information is a necessary but often insufficient condition for the adoption of preventive behaviors [11,49]. Our findings confirm this among FSWs. However, our findings also demonstrate that the indirect effects of information were significant, mediated through health behaviors and condom use skills and self-efficacy to influence condom use. Furthermore, HIV knowledge was positively associated with experiences with and attitudes toward condoms. In addition, our data show that the level of knowledge of FSWs was lower than expected, especially the level of HIV prevention knowledge. Many misconceptions about sexual transmission remained among FSWs. For example, in our sample, about half of FSWs believed that showering or washing one's genitals/private parts after sex could prevent person from becoming infected with HIV; some FSWs believed that oral sex was safe if the discharge was not swallowed. These misconceptions may result in less compliance in using a condom. Many FSWs provided correct answers about HIV knowledge concerning nonsexual activities. For example, more than 80% of the FSWs answered the item "HIV infection spreads easily when people share needles for drug use with someone" correctly. These results suggest that more attention is needed toward strengthening education about specific HIV knowledge where it is lacking.

Data in the current study confirm the role of social referents support and motivation to comply. Social referents support was positively associated with motivation to comply and experiences with and attitudes toward condoms. This finding implies that greater social referents support accompanies the strengthening of a person's motivation to comply and more positive experiences with and attitudes toward condoms. Furthermore, the direct and indirect effects of social referents support play an important role in condom use among women. Other studies among FSWs in China also found that perceived social referents support was significant with consistent condom use [22,28,50]. We did not find evidence for an association between motivation to comply and condom use while motivation to comply significantly predicted self-efficacy. When clients want or demand sex without a condom, the role of motivation to comply in FSWs' condom use decision-making may be undermined by power imbalances between FSWs and clients, fear of losing clients, and fear of being punished by employers for refusing the client's desire for sex without a condom. The results suggest that enhancing social referents support for condom use may be an important strategy to increase condom use. In addition, HIV intervention for FSWs needs to be multilevel, integrating social factors into the intervention. Encouraging a discussion about safe sex with their peers, and launching peer education among FSWs may lead to increasing levels of motivation. HIV interventions for FSWs should also include their employers. The key HIV interventions for employers include increasing their responsibility for safe sex and encouraging them to promote condom use. For instance, employers were trained to provide positive reinforcement of their employee' health sexual practices, and required to increase the availability of condoms and to promote FSWs' AIDS awareness [45,51]. Furthermore, HIV interventions should also include clients as a target population. Interventions for clients should focus on improving knowledge of HIV transmission and prevention, promoting pro-condom attitudes and behaviors, and increasing clients' access to condoms. Utilizing mass media to increase HIV knowledge, providing education concerning condom promotion for clients by sexual health services, and promoting social marketing strategy to increase clients' access to quality condoms may be effective approaches.

Perceived risk was positively associated with health behaviors and condom use skills. This result shows that the FSWs with a greater perception of risk were more likely to engage in health behaviors such as obtaining an HIV test and undergoing gynecological examinations. Perceived risk was not found to be significantly associated with the other factors. In this study, most FSWs perceived that their risk of getting infected with HIV was very low, while most FSWs had a high level of fear about getting infected with HIV. In addition, the perceived risk has a relatively low reliability estimate. These aspects may impact the associations between perceived risk and other factors. About one-third of FSWs were divorced/widowed, and so were the sole supporters of their families. Insistence on condom use could result in the loss of clients, meaning that FSWs may meet clients' demand for sex without a condom when the clients provide extra money. At the same time, lack of awareness of their high-risk status also may lead to unprotected sex. FSWs' needs for income may be more important than concerns about personal health, until clients become more accepting of condom use [52]. Therefore, HIV/AIDS interventions should increase the awareness of FSWs about HIV and should address assertiveness training to refuse unsafe sex.

When behavioral skills were divided into health behaviors and condom use skills and self-efficacy, the effects of two indicators of behavioral skills were significant while the effects of health behaviors and condom use skills were slightly stronger than the effects of self-efficacy. Self-efficacy was significantly associated with condom use, which was consistent with prior research among FSWs in China [26]. Furthermore, health behaviors and condom use skills mediated the potential effects of knowledge, perceived risk, and social referents support on condom use. These findings indicate that health behaviors and condom use skills play an important role in condom use in this sample. A few FSWs obtained an HIV test within the previous year, which is consistent with a recent study about FSWs in China [50]. Approximately 40% of FSWs reported that they had never had a gynecological examination in the last year. Previous studies reported that FSWs in China associate physical appearance with their health status [53,54]. Frequent unprotected sex is more likely to result in reproductive tract infections and sexually transmitted diseases in FSWs. Therefore, an awareness of the importance of engaging in health-promoting behaviors should also be developed in FSWs.

This study revealed that an indirect effect of substance use on condom use was significant and that lower levels of substance use accompanied positive experiences with and attitudes toward condom. In addition, substance use significantly affected self-efficacy. Higher levels of substance use may enhance intentions to seek sexual sensation and engage in high-risk sexual behavior, and express more negative condom attitudes [34,35,55-57]. Substance use may impair FSWs' mental judgment, inhibition, and the ability to negotiate condom use [56-59]. It is also possible that financial need to purchase drugs would place FSWs in a less favorable negotiating position for condom use with their clients [59]. These may lead to that FSWs with higher levels of substance use have lower self-efficacy for condom use. The current study did not find a significant relationship between substance use and health behaviors and condom use skills. Substance use may not be relevant to health behaviors that may be influenced by HIV knowledge, perceived risk, discrimination, etc. [38,60]. This would decrease the salience of health behaviors and condom use skills associated with substance use. The results suggest that future interventions should strengthen FSW understanding about the hazards of drug abuse and alcohol intoxication and reduce substance use risk behaviors among FSWs.

However, there are potential limitations to our study that suggest some caution when drawing conclusions or making generalizations from our findings. A potential limitation to this study is the cross-sectional research design that limits the ability to establish causal associations.

RDS was applied in this study to collect data among FSWs. Most studies about FSWs in China have been based on institutionalized subjects or on women working in entertainment or personal service sectors, where it is extremely difficult to obtain random samples [1]. RDS has seldom been used to study FSWs in China [61]. The financial incentive that motivates participation may not be suitable or be too little for high-income FSWs. Therefore, FSWs who earned a high income were seldom recruited [62], which may affect the representative of the sample. Additionally, RDS yields a relatively unbiased sample. Hence, our sample is an approximate representative of FSWs in Jinan.

Another limitation of this study is the self-reported nature of the questionnaire. Despite a data collection protocol designed to minimize this potential limitation, we recognize that self-reporting bias may exist. The reliability of study participant responses may be questionable due the sensitive nature of responses about sexual practices, substance use, etc. Some constructs of the IMB model in this study have relatively low reliability estimates (*e.g*., 0.56 for perceived risk, 0.61 for substance use), from which it is likely that this would have served to underestimate associations between constructs of the IMB model.

## Conclusions

The specific elements of the IMB model that are critical for condom use among FSWs were identified and confirmed the central proposition that information and motivation works mainly through behavioral skills to influence HIV prevention behaviors. The relationships between constructs of the IMB model were also examined. Our findings have implications for the development of HIV risk reduction interventions for FSWs. Further study should explore effective interventions based on the current study's findings.

## Abbreviations

FSWs: female commercial sex workers; IMB: Information-Motivation-Behavioral Skills; RDS: respondent-driven sampling; HIV: human immunodeficiency virus; STI: sexual transmitted infection; AIDS: acquired immunodeficiency syndrome; SEM: structural equation modeling; CFI: the comparative fit index; RMSEA: the Root Mean Square Errors of Approximation; NNFI: the Non-Normed Fit Index; CFA: initial confirmatory factor analysis; SD: standard deviation; M: Mean; FL: factor loadings.

## Competing interests

The authors declare that they have no competing interests.

## Authors' contributions

All authors contributed the design of this research. HZ conducted field study, collated the data, conducted statistical analyses, and prepared the first draft of the manuscript. BJ conceptualized the study, provided statistical support to the study and edited the manuscript. DK coordinated all research activities, edited the manuscript. ML provided statistical support to the study. JN, RJ, CW, SR, CZ, and XT played a major role in the field survey. All authors read and approved the final manuscript.

## Pre-publication history

The pre-publication history for this paper can be accessed here:

http://www.biomedcentral.com/1471-2458/11/113/prepub
